# Stress, Glucocorticoids and Bone: A Review From Mammals and Fish

**DOI:** 10.3389/fendo.2018.00526

**Published:** 2018-09-10

**Authors:** Paula Suarez-Bregua, Pedro Miguel Guerreiro, Josep Rotllant

**Affiliations:** ^1^Institute of Marine Research, Spanish National Research Council (IIM–CSIC), Vigo, Spain; ^2^Center of Marine Sciences (CCMAR), University of Algarve, Faro, Portugal

**Keywords:** glucocorticoids, stress, bone, vertebrates, PTH3, PTHLH

## Abstract

Glucocorticoids (GCs) are the final effector products of a neuroendocrine HPA/HPI axis governing energy balance and stress response in vertebrates. From a physiological point of view, basal GC levels are essential for intermediary metabolism and participate in the development and homeostasis of a wide range of body tissues, including the skeleton. Numerous mammalian studies have demonstrated that GC hormones exert a positive role during bone modeling and remodeling as they promote osteoblastogenesis to maintain the bone architecture. Although the pharmacological effect of the so-called stress hormones has been widely reported, the role of endogenous GCs on bone mineral metabolism as result of the endocrine stress response has been largely overlooked across vertebrates. In addition, stress responses are variable depending on the stressor (e.g., starvation, predation, and environmental change), life cycle events (e.g., migration and aging), and differ among vertebrate lineages, which react differently according to their biological, social and cognitive complexity (e.g., mineral demands, physical, and psychological stress). This review intends to summarize the endogenous GCs action on bone metabolism of mammals and fish under a variety of challenging circumstances. Particular emphasis will be given to the regulatory loop between GCs and the parathyroid hormone (PTH) family peptides, and other key regulators of mineral homeostasis and bone remodeling in vertebrates.

## Introduction

Glucocorticoids (GCs) are central steroid hormones on endocrine stress response modulation and whole-body homeostasis in vertebrates. Downstream of the hypothalamic-pituitary-adrenal/interrenal (HPA/HPI) axis, regulated by a negative feedback loop, circulating CGs exert diverse actions by binding to glucocorticoid receptor (GR) placed on nearly every tissue in the body (1). In addition to well-known effects on glucose metabolism, immune system, reproduction, feeding, circadian rhythm, behavior, and cognition, GCs also regulate bone metabolism (2–4). Bone is a metabolically active tissue, shaped at an early stage of development and continuously remodeled throughout an animals' lifetime. Bone remodeling regulated by systemic hormones, neural, and local factors, involves the coupled action of osteoclasts, osteoblasts, and osteocytes to replace old and damaged bone. This process preserves the mechanical strength and stiffness of the skeleton, maintains calcium-phosphorus homeostasis, acid/base balance, and releases growth factors as well as organic material embedded in bone (5, 6).

In vertebrates, the GCs action is complex. Despite stress hormones have long been considered as catabolic hormones, a dual metabolic effect has been found in the skeleton. Physiological levels of GCs are vital for normal skeletogenesis and bone mass accrual, which highlights an important anabolic role (7). However, an increase of GCs over the basal levels causes reduced bone growth, bone resorption and bone mineral loss as seen in Cushing's syndrome and GCs-induced osteoporosis (GIO), as well as other associated pathologies such as diabetes or sarcopenia (8–10). In humans, Cushing's syndrome (also named hypercortisolism) is characterized by an increased production of endogenous cortisol or GCs drugs resulting in detrimental effects on bone metabolism (11). Patients suffering from Cushing's disease exhibit a reduced bone mineral density, increased risk of fracture, suppression of osteoblastic differentiation and apoptosis of both osteoblasts and osteoclasts, among other symptoms (12, 13). Moreover, sustained exposure to exogenous GCs is also responsible for the so-called GIO as a consequence of long-term GC therapy (14). GIO has recently been investigated in fish, with zebrafish incubated in GCs showing reduced bone growth and impaired bone regeneration (15).

On the other hand, endogenous/exogenous GCs have been proposed to act as key regulators of osteocalcin expression in bone. Osteocalcin is a calcium-binding peptide synthesized by osteoblasts and osteocytes, involved in skeletal mineralization and, regulation of insulin production (16). Elevated GC levels suppress the osteoblast activity and inhibit the osteocalcin release in mammals (17). Therefore, GCs affecting bone formation also indirectly cause changes in whole-body energy metabolism (8). GCs are known to interact with parathyroid hormone (PTH) family members. Human PTH1 (PTH—the master regulator of bone mineral homeostasis) showed corticotropic activity in adrenocortical cell cultures (18). A feedback regulatory loop between cortisol and PTH3 (parathyroid hormone like hormone—PTHLH) has been described in vertebrates (18–20). In mammals, PTH3 participates in embryonic skeletal development (21), calcium mobilization during fetal-placental transport (22) and lactation (23, 24). While in fish, duplicated Pth3 factors are hormones involved in calcium uptake (25, 26), mineral release from scales (27), skeletogenesis and early mineralization (28).

To date, a substantial body of research has focused on the bone effects caused by a pathological increase of endogenous and exogenous GC levels, but few studies have reported the changes produced on bone metabolism due to the elevation of stress-induced GCs. As a natural mechanism, all organisms react to extrinsic and intrinsic stressors through the GC-mediated hormonal response to restore the equilibrium and preserve homeostasis. In this context, the skeleton is one of the target organs of the stress hormones and bone remodeling is an essential process that enables it to respond to changing conditions by modifying its structure and mineral composition. Stress responses are characterized by being variable across vertebrates and they are closely related to the type of stressor as well as the lineage-specific biology and ecology (29, 30). In this article, we review the action of stress-induce GCs on bone metabolism in vertebrates. Briefly, we define the current knowledge on the effect of endogenous GCs on bone under normal physiological conditions. Then, we describe how several stress factors affect bone mineral metabolism in two different vertebrate lineages: mammals (primarily human), which are endothermic terrestrial vertebrates, and fish, characterized as ectothermic aquatic vertebrates.

## Endogenous GCs on bone development and homeostasis

Endogenous GC hormones regulate the expression of target genes through GR signaling within bone cells, affecting skeletal development and metabolism. The skeleton responsiveness to GCs and the subsequent activation or inhibition of the gene expression depends on the level of circulating stress hormones, the intracellular availability of active GCs and the GR activity (1). To date the study of GC actions on bone has focused on mammalian models. Initially, investigations were based on the global GR deletion which led to premature death in newborn mice by respiratory failure (31). This was followed by more advanced molecular approaches such as the bone cell-specific GR gene deletion or the osteoblasts-targeted transgenic expression of 11βHSD2 (enzyme that catalyzes the conversion of active to inactive GCs) to disrupt intracellular GC signaling. These studies contributed to better define the endogenous GCs effects under various physiological conditions. *In vivo* and *in vitro* studies carried out in cell cultures derived from 11βHSD2 overexpressing transgenic mice have reported the positive action of endogenous GCs during bone development (32, 33). GCs appeared to be essential for mice osteoblastogenesis as they control the lineage commitment of mesenchymal progenitor cells through osteoblasts by promoting the activation of Wnt signaling. In turn, Wnt proteins act on mesenchymal cells to increase the expression of β-catenin and RUNX2, the master regulator of osteoblast differentiation. Also, osteoblast GC activity disruption in 11βHSD2 transgenic mice revealed an important role for normal intramembranous ossification and proper cartilage removal during cranial development (34, 35). In addition to the GC actions during skeletogenesis in mammals, several studies have pointed out that endogenous GCs are also required to maintain the bone mass accrual and skeletal integrity across adulthood. Inactivation of osteoblast-specific GC signaling by using a GR knockout mouse model (36) or 11βHSD2 expressing transgenic mice (37, 38) resulted in a decrease of bone mineral density in adults, which was dependent on the skeletal site and sexual maturity (37). Moreover, a downregulation in the expression of osteoblasts differentiation markers (i.e*., Col1a1, Runx2*, bone sialoprotein, and osteocalcin) was found, suggesting failed osteoblastogenesis as well as mature osteoblast function (36, 38). Therefore, the major effects of endogenous GCs on bone development and homeostasis are probably due to its direct actions on osteoblasts. Nevertheless, due to a close and reciprocal interconnectivity between osteoblasts and osteoclasts for skeletal metabolism, *in vivo* studies involving endogenous GCs and osteoclasts are needed to specifically dissect the cellular actions on the skeleton.

## Stress-induced glucocorticoid effects on bone mineral metabolism

**Figure 1:**
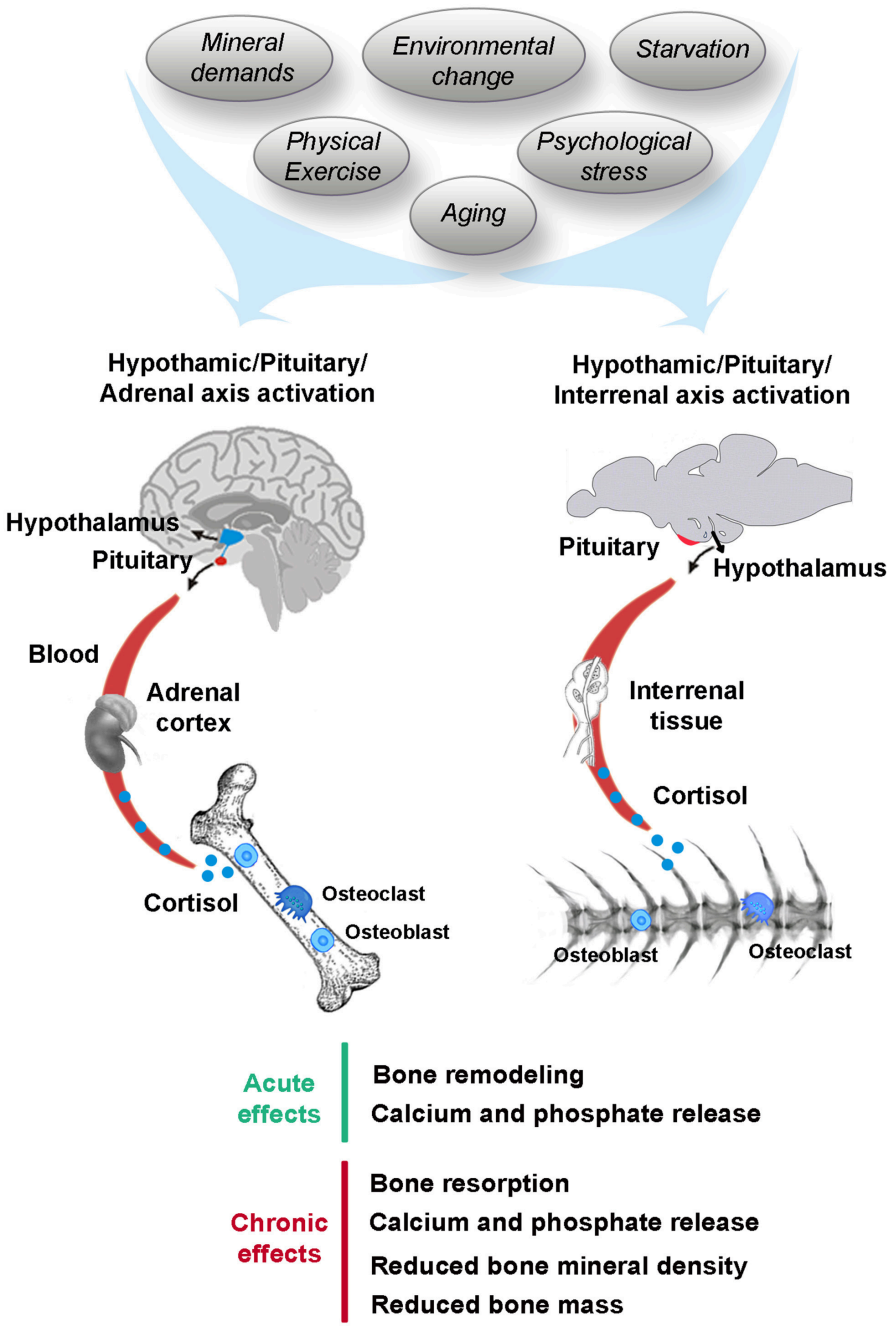
Stress-induced cortisol affecting bone mineral metabolism in human and fish. Stressors including mineral demands, environmental change, starvation, physical exercise, psychological stress, or aging trigger the hypothalamic-pituitary-adrenal/interrenal (HPA/HPI) axis activation in human and fish, respectively, leading acute and chronic effects on the skeleton.

GCs, including cortisol and/or corticosterone in mammals as well as cortisol in fish, are synthesized in the adrenal cortex of mammals, but in the interrenal tissue of the head kidneys in teleosts (39). In response to stress, the pituitary gland signals the adrenal gland/interrenal tissue to release GCs. These GCs are released into the blood and initiate numerous cellular events that promote changes in cells and tissues for adaptation to stressful stimuli (40) (Figure 1). In this context, it is important to distinguish between the degrees of stress that can ultimately affect bone homeostasis. Acute stress is sudden and transitory and it may trigger skeletal remodeling as an adaptive response, which confers survival advantage (41). After exposure to an acute stressor, GCs levels are rapidly increased in the blood before returning to basal levels via negative feedback mechanisms. However, chronic stress is a long-term stressor, sustained for a prolonged period of time or due to a frequently occurring stressor (41), through which GCs levels remain elevated which could lead to several pathological conditions including bone mineral loss (2). Stress-induced bone resorption can result in calcium and phosphate release and it can lead to irreversible damage of the bone architecture resulting in mechanical instability. In addition to intensity and duration of the stressor, the stress responses of vertebrates are highly variable depending on the type of stressor and the way it is perceived by each kind of species. Some key stress factors affecting bone mineral metabolism in mammals and fish are described in this section including mineral demands, environmental change, starvation, physical exercise, psychological stress, and aging (Figure 1).

### Mineral demands

The skeleton is the major mineral storage organ in the vertebrate body and takes part in the regulation of calcium-phosphate metabolism. Thus, skeleton provides calcium and phosphate through bone resorption to compensate the inadequate availability of minerals in the environment and/or in the diet to maintain essential ionic levels in blood (5, 42). Unlike terrestrial vertebrates, fish can absorb minerals from surrounding water across the skin, oral and branchial epithelium, so stressors related to water and ion homeostasis have a greater physiological impact (29). In teleosts, the role of cortisol on osmoregulation has widely been reported (43) but, the contribution of cortisol on the ionic balance related to bone mineral homeostasis has received less attention (44). Previous studies showed that fish exposed to low calcium water levels give rise to high plasma cortisol levels in rainbow trout (45, 46), and stimulates the gene expression of steroid 11β-hydroxylase (final-step enzyme for cortisol synthesis) as well as glucocorticoid receptor (*gr*) in zebrafish (47). Moreover, cortisol treatment was shown to induce *in vitro* calcium transport in cultured rainbow trout gill epithelium, which supports its hypercalcemic role (48). Also, tilapia exposed to exogenous cortisol showed an increase in calcium uptake and upregulation of epithelial Ca^2+^ channel (*ecac*) gene expression (49). It would therefore appear that teleost fish regulate the calcium uptake to cope with a fluctuating water environment which is closely related to bone homeostasis. Alternatively, studies with juvenile seabream showed a plasma cortisol increase after prolonged exposure to low calcium availability in the water and/or diet, which resulted in reduced whole-body calcium and phosphorus contents (50). In the European eel, chronic cortisol treatment induced mineral loss in vertebral bone through osteoclastic resorption and osteocytic osteolysis (51). Interestingly, it has been suggested that cortisol mobilization of bone mineral stores in eel may be evidence of an ancestral stress-induced physiological process (51) related to the effects of stress events in mammals (e.g., starvation, physical exercise, psychological stress, or aging).

An interaction between hypercalcemic PTH factors regulating bone mineral metabolism and cortisol has been reported in mammals and fish (Table 1). Both PTH1 and PTH3, stimulated cortisol release from human adrenocortical cells *in vitro* (18), although only the gene encoding PTH3 appears to be regulated by GCs (19). Similarly, piscine Pth3 showed *in vitro* corticotropic activity on isolated sea bream interrenal glands (20). In turn, sustained cortisol levels in sea bream as a consequence of a 24h confinement stressor or *in vivo* cortisol intraperitoneal injection resulted in a decrease in plasma Pth3 levels (52). Similar to cortisol, sea bream PTH3 is produced in interrenal tissue in fish (20, 53) and therefore an autocrine and/or paracrine regulatory mechanism between these two hormones was proposed (52). However the underlying molecular regulation remains unclear and it is possible that Pth3 acts indirectly at other levels of the HPI axis. Contradictory results regarding the cortisol-Pth3 reciprocal regulation were found in sea bream exposed to limited calcium availability in the long-term. Fish either under low calcium water along with a calcium-sufficient diet or under regular calcium water but calcium-deficient diet showed elevated plasma cortisol and Pth3 levels (50).

**Table 1:** Summary of some of the reported studies including PTH-cortisol regulatory interactions in mammals and fish.

**Hormone**	**Species**	**Action**	**Tissue**	**References**
PTH1	*Homo sapiens*	Cortisol release	Adrenocortical cells culture	18
PTH3	*Homo sapiens*	Cortisol release	Adrenocortical cells culture	18
Cortisol	*Mus musculus*	PTH3 expression increase	Kidney	19
Pth3	*Sparus aurata*	Cortisol release	Isolated interrenal glands	20
Cortisol	*Sparus aurata*	Pth3 expression decrease	Blood	52

### Environmental change

Environmental stressors like temperature fluctuations are a critical feature of homeostasis in an organism. This is of particular relevance for ectothermic animals such as fish, where temperature directly influences their normal physiology. Sea bream exposed to water temperatures below 13°C develop winter syndrome, which is characterized by a multi-organ dysfunction together with a high but transient rise of plasma cortisol levels triggering a stress response (54, 55). A recent study in sea bream has revealed the impact of cold challenge, which increased the cortisol production and affected bone homeostasis in juveniles (55). Thus, fish exposed to low temperature during early development showed altered enzymatic activities of alkaline phosphatase (ALP) and tartrate-resistant acid phosphatase (TRAP) as well as calcium content changes on the vertebral bone (55). Interestingly, temperature is also a modulator of the expression of PTH family members. Zebrafish embryos exposed to cold (18°C) stress showed up-regulated mRNA levels of *pth1a, pth1b, pth3a, pth3b*, and *pth1rb*, while those exposed to a hot (38°C) stress down-regulated mRNA levels of these genes (56). Therefore, it is likely that such changes may impact mineral balance, altering bone development in embryos. However, to our knowledge, there are no studies showing a correlation between temperature-driven levels of cortisol and PTH family members affecting bone metabolism.

### Starvation

A common stressor in the wild is food deprivation, which can be caused by adverse weather, decline in prey availability, increased predator pressure and migration or hibernation, among others. Under these conditions, it is well know that GCs are released into the blood to promote the mobilization and utilization of energy reserves and mineral stores in vertebrates (57). Regarding migratory teleost fish like salmonids, spawning migration is a very challenging situation since they undergo not only fasting but also exhausting exercise, changes in osmoregulation and sexual maturation (58). Thus, migratory salmonids, essentially as adults returning to spawning grounds, experience a strong activation of the neuroendocrine axis resulting in elevated plasma corticosteroid levels (59) as well as marked resorption of the skeleton. In particular, the anadromous Atlantic salmon was reported to experience a dramatic skeletal transformation caused by a decrease in the bone mineral content, halastic demineralization, osteoclastic resorption, and reduced vertebral bone mass (60–62). Nevertheless, a recent study in the migratory European eel showed that sexually mature fish via cortisol injection exhibited severe bone loss in the vertebrae and skull, while plasma cortisol levels were reduced (63). Therefore, a cortisol-independent bone resorption mechanism has been suggested in migratory eels (63). Some mammalian species also experience a situation of nutritional deprivation during hibernation similar to that observed in migratory fish. Small mammals such as little brown bats and hamsters lose a significant bone mineral volume during hibernation (64, 65), but only high plasma cortisol levels have been detected in bats (66). On the other hand, cortisol is increased in hibernating bears, however they maintain a typically balanced bone turnover which prevents bone reabsorption excess and osteoporosis (67, 68). Furthermore, fasting studies in humans have shown an increase in blood cortisol concentration (69) accompanied by a decrease of PTH secretion, which is suggested to have some positive effect on the bone health (70).

### Physical exercise

Physical exercise represents a stressful experience for all organisms. In mammals, physical activity promotes direct effects on bone metabolism via mechanical forces (i.e., weight-bearing activities), but also indirectly through hormonal factors (71). Hence, exercise causes HPA axis activation and the subsequent release of GCs into the blood. Although physical exercise has been reported to prevent bone mineral loss and to sustain bone health, long-term intense exercise is reported to cause hypercortisolism, which can result in osteopenia and osteoporosis (71). Some studies have showed that over-trained runners exhibit elevated ACTH and cortisol basal concentrations compared with moderately trained runners and sedentary subjects (72). However, the HPA axis activation was attenuated in over-trained runners after exposure to an acute exercise, suggesting a certain adaptation to physical exercise (72). Other investigations have reported that highly trained male master cyclists (73) and competitive male cyclists show low bone mineral density in the hip and spine, however there is no clear association between bone mineral content and excess of GC secretion (74). Exercised fish show improved growth and increased bone remodeling (75). However, the most extreme examples of possible interactions between GCs and bone metabolism during exercise may arise from migratory fish such as the salmonids or eels (see also under *Aging*). In experiments that were aimed to simulate to some extent the skeletal-loss consequences of a 5,000 km migration to reproductive grounds (51, 63) demonstrated that cortisol induced a significant bone demineralization of Europen eel vertebrae, with significant decreases of the mineral ratio and the degree of mineralization of vertebral sections. Using histology and image analysis of ultrathin microradiographs they showed the induction by cortisol of different mechanisms of bone resorption, including periosteocytic osteolysis and osteoclastic resorption. These effects were further enhanced by sex steroids. Specificity of cortisol action was investigated by comparison with the effects of sex steroids, namely estradiol, related to the stimulated synthesis of vitellogenin (Vg), an oviparous specific phospho-calcio-lipoprotein. Such effects of estradiol have been profusely shown in salmonids (76). However, in above study, the ready-to-migrate eels were not actually exercised but simply injected with steroids and thus the evidence for the effects of exercise-related GCs.

### Psychological stress

It has recently been demonstrated that psychological stress affects bone metabolism in humans and some animal models (77–81). Although the psychological stress response is complex, as it depends on individual interpretation, it has been suggested that long-term psychological stress produces altered HPA axis activity and as a consequence, GC release affecting bone health (77). In rats, chronic psychological stress by anxiety neurosis results in the loss of mandibular bone matrix (78). Post-traumatic stress disorder, which is related to altered serum GCs, caused a decrease of bone mineral density and bone mineral contents in young mice (79). In humans, the relationship between depression and bone mineral density has also been associated with stress-induced cortisol effects. Post-menopausal women with depression showed loss of bone mineral density in the lumbar spine and femur compared to non-depressed subjects, as well as a higher cortisol production after an acute stress experience (80). Furthermore, pre-menopausal women suffering from chronic depression presented a negative correlation between cortisol levels and bone mineral density, as well as low osteocalcin levels suggesting a decrease in bone formation (81). Recently there has been increased attention to the impact of social or psychological stress in fish, in parallel with the recognition of an increased degree of sentience and multiple individual coping styles, to which some may even refer as “personalities” in fish. The way fish exhibiting those different coping styles address stressful events determines to some extent their rank, access to food, energy expenditure, growth rates and cortisol response levels (82, 83). However, to date, there is no information on the impact of psychological stress and induced GC levels on fish bone.

### Aging

Aging is an imbalance between damage and repair that makes organisms undergo an increasing vulnerability to challenges during the post-maturational life, decreasing their ability to survive (84). Along these lines, aging disturbs the homeostatic system but perhaps it should not be considered as a stressor since it does not elicit *per se* a physiological stress response. However, aging is closely related to responsivity to stress and it seems to produce similar effects to those seen in the chronic stress response. In mammals, aging causes greater HPA axis activation and thereby an excess production of GCs that negatively affect bone metabolism (7). It has been proposed that HPA axis hyperactivity could be due to a decrease in the number of GC receptors in the brain, which in turn affects the negative feedback regulation, but can also be the result of repeated stress events (7). An age-related increase of corticosterone as well as upregulation of 11βHSD1 (enzyme that activates GCs) expression in bone, which led to reduced bone vasculature and skeletal fragility in mice (85). Studies in humans have provided evidence that elevated cortisol levels affect bone mineral density. Thus, elderly men and women with a high level of evening salivary cortisol had a reduced bone mineral density in the lumbar spine (86). Also, high plasma cortisol levels in older women contributed to bone loss in the femoral neck (87). Additionally, a positive correlation between cortisol concentration and bone loss rate was found in the lumbar spine in elderly men (88). Fish grow continuously throughout their lives and usually their skeleton maintains its integrity with aging. A few exceptions can be found in semelparous species, such as many salmonids and eels (51, 63, 76) in which sexual maturation, reproduction and related skeletal remodeling coincide with the end of life. Both GC and sex steroids increase along the migratory route and peak levels coincide with important organ and skeletal remodeling. In pink salmon specifically, cortisol levels rise over 20-fold in both males and females (89) being thus likely that GCs may have important effects over bone metabolism. Despite the fact that most fish do not appear to undergo important skeletal changes as they age, the use of fish as models for probing into aging-related health conditions with impacts on bone mineral metabolism in human offers ample possibilities, since they can be treated and selected to simulate such conditions, including those directly or indirectly related to disturbances in circulating GCs (90–93).

## Conclusion

In response to a variety of stressful situations and/or stimuli that challenge the internal equilibrium in vertebrates, bone appears to be a target organ for stress-induced GCs produced by HPA/HPI axis activation. In mammals, as in fish, elevated GC levels sustained over time result in bone resorption, which alters the mineral balance and damages the bone structure. Although this evidence suggests that stress-induced GCs may act in a similar fashion to that of therapeutic GCs, there is a gap in the knowledge about the cellular and molecular mechanisms involving the stress response, cortisol and bone mineral metabolism in vertebrates. Studies utilizing mammalian models based on the pathological increase of endogenous GCs and pharmacological GCs reported that the bone effect of these hormones could be due to its direct action on osteoblasts (34, 35). However, the actions of stress-induced GCs on bone cells as well as the interactions between GCs and other factors regulating bone homeostasis are currently unknown.

## Author contributions

PS-B, PG, and JR wrote and revised the manuscript.

### Conflict of interest statement

The authors declare that the research was conducted in the absence of any commercial or financial relationships that could be construed as a potential conflict of interest.

## References

[1] Moutsatsou P Kassi E Papavassiliou AG. Glucocorticoid receptor signaling in bone cells. Trends Mol Med. (2012) 18:348–59. 10.1016/j.molmed.2012.04.005 22578718

[2] Sapolsky RM Romero LM Munck AU. How do glucocorticoids influence stress responses? Integrating permissive, suppressive, stimulatory, and preparative actions. Endocr Rev. (2000) 21:55–89. 10.1210/er.21.1.55 10696570

[3] Hartmann K Koenen M Schauer S Wittig-Blaich S Ahmad M Baschant U. Molecular actions of glucocorticoids in cartilage and bone during health, disease, and steroid therapy. Physiol Rev. (2016) 96:409–47. 10.1152/physrev.00011.2015 26842265

[4] Subramaniam M Colvard D Keeting PE Rasmussen K Riggs BL Spelsberg TC. Glucocorticoid regulation of alkaline phosphatase, osteocalcin, and proto-oncogenes in normal human osteoblast-like cells. J Cell Biochem. (1992) 50:411–24. 10.1002/jcb.240500410 1469072

[5] Kini U Nandeesh BN Physiology of bone formation, remodeling, and metabolism. In: Fogelman I Gnanasegaran G Wall H, editors. Radionuclide and Hybrid Bone Imaging. Berlin; Heidelberg: Springer Berlin Heidelberg (2010) p. 29–57.

[6] Dempster D Anatomy and functions of the adult skeleton. In: FFavus M, editor. Primer on the Metabolic Bone Diseases and Disorders of Mineral Metabolism. Washington, DC: American Society for Bone and Mineral Research (2006) p. 7–11.

[7] Zhou H Cooper MS Seibel MJ. Endogenous glucocorticoids and bone. Bone Res. (2013) 1:107–19. 10.4248/BR201302001 26273496 PMC4472112

[8] Henneicke H Gasparini SJ Brennan-Speranza TC Zhou H Seibel MJ. Glucocorticoids and bone: local effects and systemic implications. Trends Endocrinol Metabol. (2014) 25:197–211. 10.1016/j.tem.2013.12.006 24418120

[9] Gonzalez-Gonzalez JG Mireles-Zavala LG Rodriguez-Gutierrez R Gomez-Almaguer D Lavalle-Gonzalez FJ Tamez-Perez HE. Hyperglycemia related to high-dose glucocorticoid use in noncritically ill patients. Diabetol Metabol Syndrome (2013) 5:18. 10.1186/1758-5996-5-18 23557386 PMC3635995

[10] Kanda F Okuda S Matsushita T Takatani K Kimura K Chihara K. Steroid myopathy: pathogenesis and effects of growth hormone and insulin-like growth factor-I administration. Hor Res Paediatr. (2001) 56:24–8. 10.1159/000048130 11786681

[11] Wagner-Bartak NA Baiomy A Habra MA Mukhi SV Morani AC Korivi BR. Cushing syndrome: diagnostic workup and imaging features, with clinical and pathologic correlation. Am J Roentgenol. (2017) 209:19–32. 10.2214/AJR.16.17290 28639924

[12] Mancini T Doga M Mazziotti G Giustina A. Cushing's syndrome and bone. Pituitary (2004) 7:249–52. 10.1007/s11102-005-1051-2 16010458

[13] Kaltsas G Makras P. Skeletal diseases in cushing's syndrome: osteoporosis versus arthropathy. Neuroendocrinology (2010) 92:60–4. 10.1159/000314298 20829620

[14] Mazziotti G Angeli A Bilezikian JP Canalis E Giustina A. Glucocorticoid-induced osteoporosis: an update. Trends Endocrinol Metabol. (2006) 17:144–9. 10.1016/j.tem.2006.03.009 16678739

[15] Geurtzen K Vernet A Freidin A Rauner M Hofbauer LC Schneider JE. Immune suppressive and bone inhibitory effects of prednisolone in growing and regenerating zebrafish tissues. J Bone Mineral Res. (2017) 32:2476–2488. 10.1002/jbmr.3231 28771888

[16] Fernández-Real JM Izquierdo M Ortega F Gorostiaga E Gómez-Ambrosi J Moreno-Navarrete JM. The relationship of serum osteocalcin concentration to insulin secretion, sensitivity, and disposal with hypocaloric diet and resistance training. J Clin Endocrinol Metabol. (2009) 94:237–45. 10.1210/jc.2008-0270 18854399

[17] Brennan-Speranza TC Henneicke H Gasparini SJ Blankenstein KI Heinevetter U Cogger VC. Osteoblasts mediate the adverse effects of glucocorticoids on fuel metabolism. J Clin Invest. (2012) 122:4172–89. 10.1172/JCI63377 23093779 PMC3484445

[18] Mazzocchi G Aragona F Malendowicz LK Nussdorfer GG. PTH and PTH-related peptide enhance steroid secretion from human adrenocortical cells. Am J Physiol Endocrinol Metabol. (2001) 280:E209–13. 10.1152/ajpendo.2001.280.2.E209 11158922

[19] Yoo YM Baek MG Jung EM Yang H Choi KC Yu FH. Parathyroid hormone-related protein and glucocorticoid receptor beta are regulated by cortisol in the kidney of male mice. Life Sci. (2011) 89:615–20. 10.1016/J.LFS.2011.08.001 21872610

[20] Rotllant J Guerreiro PM Anjos L Redruello B Canario AVM Power DM. Stimulation of cortisol release by the N terminus of teleost parathyroid hormone-related protein in interrenal cells *in vitro*. Endocrinology (2005) 146:71–6. 10.1210/en.2004-0644 15459121

[21] Kronenberg HM. PTHrP and skeletal development. Ann N Y Acad Sci. (2006) 1068:1–13. 10.1196/annals.1346.002 16831900

[22] Kovacs CS Lanske B Hunzelman JL Guo J Karaplis AC Kronenberg HM. Parathyroid hormone-related peptide (PTHrP) regulates fetal-placental calcium transport through a receptor distinct from the PTH/PTHrP receptor. Proc Natl Acad Sci USA. (1996) 93:15233–8. 8986793 10.1073/pnas.93.26.15233 PMC26386

[23] Neville MC McFadden TB Forsyth I. Hormonal regulation of mammary differentiation and milk secretion. J Mammary Gland Biol Neoplasia (2002) 7:49–66. 10.1023/A:1015770423167 12160086

[24] VanHouten J Dann P McGeoch G Brown EM Krapcho K Neville M Wysolmerski JJ. The calcium-sensing receptor regulates mammary gland parathyroid hormone-related protein production and calcium transport. J Clin Invest. (2004) 113:598–608. 10.1172/JCI18776 14966569 PMC338258

[25] Abbink W Bevelander GS Hang X Lu W Guerreiro PM Spanings T. PTHrP regulation and calcium balance in sea bream (*Sparus auratus* L.) under calcium constraint. J Exp Biol. (2006) 209:3550–7. 10.1242/jeb.02399 16943495

[26] Guerreiro PM Fuentes J Power DM Ingleton PM Flik G Canario AV. Parathyroid hormone-related protein: a calcium regulatory factor in sea bream (*Sparus aurata L*.) larvae. Am J Physiol Regulat Integr Comp Physiol. (2001) 281:R855–60. 10.1152/ajpregu.2001.281.3.R855 11507001

[27] Rotllant J Redruello B Guerreiro PM Fernandes H Canario AVM Power DM. Calcium mobilization from fish scales is mediated by parathyroid hormone related protein via the parathyroid hormone type 1 receptor. Reg Peptides (2005) 132:33–40. 10.1016/j.regpep.2005.08.004 16181689

[28] Yan YL Bhattacharya P He XJ Ponugoti B Marquardt B Layman J. Duplicated zebrafish co-orthologs of parathyroid hormone-related peptide (PTHrP, Pthlh) play different roles in craniofacial skeletogenesis. J Endocrinol. (2012) 214:421–35. 10.1530/JOE-12-0110 22761277 PMC3718479

[29] Wendelaar Bonga SE The stress response in fish. Physiol Rev. (1997) 77:591–625. 10.1152/physrev.1997.77.3.591 9234959

[30] Reeder DM Kramer KM Stress in free-ranging mammals: integrating physiology, ecology, and natural history. J Mammal. (2005) 86:225–35. 10.1644/BHE-003.1

[31] Cole TJ Blendy JA Monaghan AP Krieglstein K Schmid W Aguzzi A. Targeted disruption of the glucocorticoid receptor gene blocks adrenergic chromaffin cell development and severely retards lung maturation. Genes Dev. (1995) 9:1608–21. 7628695 10.1101/gad.9.13.1608

[32] Zhou H Mak W Zheng Y Dunstan CR Seibel MJ. Osteoblasts directly control lineage commitment of mesenchymal progenitor cells through Wnt signaling. J Biol Chem. (2008) 283:1936–45. 10.1074/jbc.M702687200 18045882

[33] Shalhoub V Conlon D Stein GS Lian JB Tassinari M Quinn C. Glucocorticoids promote development of the osteoblast phenotype by selectively modulating expression of cell growth and differentiation associated genes. J Cell Biochem. (1992) 50:425–40. 10.1002/jcb.240500411 1469073

[34] Zhou H Mak W Kalak R Street J Fong-Yee C Zheng Y. Glucocorticoid-dependent Wnt signaling by mature osteoblasts is a key regulator of cranial skeletal development in mice. Development (2009) 136:427–36. 10.1242/dev.027706 19141672

[35] Yang M Trettel LB Adams DJ Harrison JR Canalis E Kream BE. Col3.6-HSD2 transgenic mice: a glucocorticoid loss-of-function model spanning early and late osteoblast differentiation. Bone (2010) 47:573–82. 10.1016/j.bone.2010.06.002 20541046 PMC2926146

[36] Rauch A Seitz S Baschant U Schilling AF Illing A Stride B. Glucocorticoids suppress bone formation by attenuating osteoblast differentiation via the monomeric glucocorticoid receptor. Cell Metabol. (2010) 11:517–31. 10.1016/j.cmet.2010.05.005 20519123

[37] Kalak R Zhou H Street J Day RE Modzelewski JRK Spies CM. Endogenous glucocorticoid signalling in osteoblasts is necessary to maintain normal bone structure in mice. Bone (2009) 45:61–7. 10.1016/j.bone.2009.03.673 19358901

[38] Sher LB Harrison JR Adams DJ Kream BE. Impaired cortical bone acquisition and osteoblast differentiation in mice with osteoblast-targeted disruption of glucocorticoid signaling. Calcif Tissue Int. (2006) 79:118–25. 10.1007/s00223-005-0297-z 16927049

[39] Tort L Teles M The Endocrine Response to Stress - A Comparative View. Basic and Clinical Endocrinology Up-to-Date. Rijeka: InTech 10.5772/21446

[40] Chrousos GP. Stress and disorders of the stress system. Nat Rev Endocrinol. (2009) 5:374–81. 10.1038/nrendo.2009.106 19488073

[41] Chrousos GP Gold PW. The concepts of stress and stress system disorders. Overview of physical and behavioral homeostasis. JAMA (1992) 267:1244–52. 1538563

[42] Witten PE Huysseune A. A comparative view on mechanisms and functions of skeletal remodelling in teleost fish, with special emphasis on osteoclasts and their function. Biol Rev Cambridge Philos Soc. (2009) 84:315–46. 10.1111/j.1469-185X.2009.00077.x 19382934

[43] Mommsen TP Vijayan MM Moon TW Cortisol in teleosts: dynamics, mechanisms of action, and metabolic regulation. Rev Fish Biol Fish. (1999) 9:211–68.

[44] Mccormick SD Endocrine control of osmoregulation in teleost fish. Integr Comp Biol. (2001) 41:781–94. 10.1093/icb/41.4.781

[45] Perry SF Wood CM Kinetics of branchial calcium uptake in the rainbow trout: effects of acclimation to various external calcium levels. J Exp Biol. (1985) 116:411–33.

[46] Flik G Perry SF. Cortisol stimulates whole body calcium uptake and the branchial calcium pump in freshwater rainbow trout. J Endocrinol. (1989) 120:75–82. 2521890 10.1677/joe.0.1200075

[47] Lin CH Tsai IL Su CH Tseng DY Hwang PP Reverse effect of mammalian hypocalcemic cortisol in fish: cortisol stimulates Ca 2+ uptake via glucocorticoid receptor-mediated vitamin D 3 metabolism. PLoS ONE (2011) 6:e23689 10.1371/journal.pone.0023689 21887296 PMC3161063

[48] Kelly SP Wood CM. Cortisol stimulates calcium transport across cultured gill epithelia from freshwater rainbow trout. In Vitro Cell Dev Biol Anim. (2008) 44:96–104. 10.1007/s11626-007-9077-6 18239979

[49] Lin C Kuan W Liao B Deng A Tseng D Hwang P. Environmental and cortisol-mediated control of Ca ^2+^ uptake in tilapia (*Oreochromis mossambicus*). J Comp Physiol B (2016) 186:323–32. 10.1007/s00360-016-0963-7 26857273 PMC4791471

[50] Abbink W Bevelander GS Rotllant J Canario AVM Flik G. Calcium handling in Sparus auratus: effects of water and dietary calcium levels on mineral composition, cortisol and PTHrP levels. J Exp Biol. (2004) 207:4077–84. 10.1242/jeb.01254 15498953

[51] Sbaihi M Rousseau K Baloche S Meunier F Fouchereau-Peron M Dufour S. Cortisol mobilizes mineral stores from vertebral skeleton in the European eel: an ancestral origin for glucocorticoid-induced osteoporosis? J Endocrinol. (2009) 201:241–52. 10.1677/JOE-08-0492 19223398

[52] Guerreiro PM Rotllant J Fuentes J Power DM Canario AVM. Cortisol and parathyroid hormone-related peptide are reciprocally modulated by negative feedback. Gen Comp Endocrinol. (2006) 148:227–35. 10.1016/j.ygcen.2006.03.004 16624313

[53] Rotllant J Worthington G Fuentes J Guerreiro P Teitsma C Ingleton P. Determination of tissue and plasma concentrations of PTHrP in fish: development and validation of a radioimmunoassay using a teleost 1–34 N-terminal peptide. Gen Comp Endocrinol. (2003) 133:146–53. 10.1016/S0016-6480(03)00166-7 12899855

[54] Rotllant J Balm PHM Wendelaar-Bonga SE Pérez-Sánchez J Tort L A drop in ambient temperature results in a transient reduction of interrenal ACTH responsiveness in the gilthead sea bream (*Sparus aurata*, L.). Fish Physiol Biochem. (2000) 23:265–73. 10.1023/A:1007873811975

[55] Mateus AP Costa R Gisbert E Pinto PIS Andree KB Estévez A. Thermal imprinting modifies bone homeostasis in cold-challenged sea bream (*Sparus aurata*). J Exp Biol. (2017) 220:3442–54. 10.1242/jeb.156174 28733328

[56] Jin Y Lan Z Zhu G Lu W Acute salinity and temperature challenges during early development of zebrafish: Differential gene expression of PTHs, PTHrPs and their receptors. Aquacult Fish. (2017) 2:49–58. 10.1016/J.AAF.2017.04.001

[57] Sheriff MJ Dantzer B Delehanty B Palme R Boonstra R. Measuring stress in wildlife: techniques for quantifying glucocorticoids. Oecologia (2011) 166:869–87. 10.1007/s00442-011-1943-y 21344254

[58] Mommsen TP Vijayan MM Moon TW Cortisol in teleosts: dynamics, mechanisms of action, and metabolic regulation. Rev Fish Biol Fish. (1999) 9:211–68.

[59] Carruth LL Dores RM Maldonado TA Norris DO Ruth T Jones RE. Elevation of plasma cortisol during the spawning migration of landlocked kokanee salmon (*Oncorhynchus nerka kennerlyi*). Comp Biochem Physiol Part C (2000) 127:123–31. 10.1016/S0742-8413(00)00140-7 11083023

[60] Kacem A Meunier FJ Halastatic demineralization in the vertebrae of Atlantic salmon, during their spawning migration. J Fish Biol. (2003) 63:1122–30. 10.1046/j.1095-8649.2003.00229.x

[61] Kacem A Meunier FJ Bagliniere JL A quantitative study of morphological and histological changes in the skeleton of Salmo salar during its anadromous migration. J Fish Biol. (1998) 53:1096–109. 10.1111/j.1095-8649.1998.tb00466.x

[62] Kacem A Gustafsson S Meunier FJ. Demineralization of the vertebral skeleton in Atlantic salmon *Salmo salar* L. during spawning migration. Comp Biochem Physiol Part A Mol Integr Physiol. (2000) 125:479–84. 10.1016/S1095-6433(00)00174-4 10840223

[63] Rolvien T Nagel F Milovanovic P Wuertz S Marshall RP Jeschke A. How the European eel (*Anguilla anguilla*) loses its skeletal framework across lifetime. Proc R Soc B (2016) 283:20161550. 10.1098/rspb.2016.1550 27798301 PMC5095380

[64] Steinberg B Singh IJ Mitchell OG. The effects of cold-stress, hibernation, and prolonged inactivity on bone dynamics in the golden hamster,Mesocricetus auratus. J Morphol. (1981) 167:43–51. 10.1002/jmor.1051670105 7241597

[65] Whalen JP Krook L Nunez EA. A radiographic and histologic study of bone in the active and hibernating bat (*Myotis lucifugus*). Anatom Record (1972) 172:97–107. 10.1002/ar.1091720109 5007373

[66] Gustafson AW Belt WD. The adrenal cortex during activity and hibernation in the male little brown bat, *Myotis lucifugus lucifugus*: annual rhythm of plasma cortisol levels. Gen Comp Endocrinol. (1981) 44:269–78. 10.1016/0016-6480(81)90001-0 7286611

[67] Seger RL Cross RA Rosen CJ Causey RC Gundberg CM Carpenter TO. Investigating the mechanism for maintaining eucalcemia despite immobility and anuria in the hibernating American black bear (*Ursus americanus*). Bone (2011) 49:1205–12. 10.1016/j.bone.2011.08.017 21893223

[68] Doherty AH Florant GL Donahue SW. Endocrine regulation of bone and energy metabolism in hibernating mammals. Int Comp Biol. (2014) 54:463–83. 10.1093/icb/icu001 24556365 PMC4184349

[69] Bahijri S Borai A Ajabnoor G Abdul Khaliq A AlQassas I Al-Shehri D Relative metabolic stability, but disrupted circadian cortisol secretion during the fasting month of ramadan. PLoS ONE (2013) 8:e60917 10.1371/journal.pone.0060917 23637777 PMC3630175

[70] Bahijri SM Ajabnoor GM Borai A Al-Aama JY Chrousos GP. Effect of Ramadan fasting in Saudi Arabia on serum bone profile and immunoglobulins. Therap Adv Endocrinol Metabol. (2015) 6:223–32. 10.1177/2042018815594527 26445645 PMC4579416

[71] Mastorakos G Pavlatou M Diamanti-Kandarakis E Chrousos GP. Exercise and the stress system. Hormones (2005) 4:73–89. Available online at: http://www.hormones.gr/57/article/article.html 16613809

[72] Luger A Deuster PA Kyle SB Gallucci WT Montgomery LC Gold PW. Acute hypothalamic–pituitary–adrenal responses to the stress of treadmill exercise. N Eng J Med. (1987) 316:1309–15. 10.1056/NEJM198705213162105 3033504

[73] Nichols JF Palmer JE Levy SS. Low bone mineral density in highly trained male master cyclists. Osteoporos Int. (2003) 14:644–9. 10.1007/s00198-003-1418-z 12856112

[74] Mathis SL Farley RS Fuller DK Jetton AE Caputo JL. The relationship between cortisol and bone mineral density in competitive male cyclists. J Sports Med. (2013) 2013:1–7. 10.1155/2013/896821 26464885 PMC4590890

[75] Suniaga S Rolvien T vom Scheidt A Fiedler IAK Bale HA Huysseune A. Increased mechanical loading through controlled swimming exercise induces bone formation and mineralization in adult zebrafish. Sci Rep. (2018) 8:3646. 10.1038/s41598-018-21776-1 29483529 PMC5826918

[76] Persson P Johannsson SH Takagi Y Björnsson BT Estradiol-17β and nutritional status affect calcium balance, scale and bone resorption, and bone formation in rainbow trout, *Oncorhynchus mykiss*. J Comp Physiol B (1997) 167:468–73. 10.1007/s003600050098

[77] Wippert PM Rector M Kuhn G Wuertz-Kozak K. Stress and alterations in bones: an interdisciplinary perspective. Front Endocrinol. (2017) 8:1–7. 10.3389/fendo.2017.00096 28507534 PMC5410657

[78] Neporada KS Leont'eva FS Tarasenko LM. Chronic stress impairs structural organization of organic matrix in bone tissue of rat periodontium. Byulleten' Eksperimental'noi Biologii i Meditsiny (2003) 135:637–8. 10.1023/A:1025464932135 12937668

[79] Yu H Watt H Kesavan C Johnson PJ Wergedal JE Mohan S. Lasting consequences of traumatic events on behavioral and skeletal parameters in a mouse model for post- Traumatic Stress Disorder (PTSD). PLoS ONE (2012) 7:e42684. 10.1371/journal.pone.0042684 22927935 PMC3425500

[80] Furlan PM Ten Have T Cary M Zemel B Wehrli F Katz IR. The role of stress-induced cortisol in the relationship between depression and decreased bone mineral density. Biol Psychiatry (2005) 57:911–7. 10.1016/J.BIOPSYCH.2004.12.033 15820712

[81] Altindag O Altindag A Asoglu M Gunes M Soran N Deveci Z. Relation of cortisol levels and bone mineral density among premenopausal women with major depression. Int J Clin Pract. (2007) 61:416–20. 10.1111/j.1742-1241.2006.01276.x 17313608

[82] Sørensen C Johansen IB Øverli Ø. Neural plasticity and stress coping in teleost fishes. Gen Compar Endocrinol. (2013) 181:25–34. 10.1016/j.ygcen.2012.12.003 23274407

[83] Castanheira MF Conceição LEC Millot S Rey S Bégout M-L Damsgård B Coping styles in farmed fish: consequences for aquaculture. Rev Aquacult. (2017) 9:23–41. 10.1111/raq.12100

[84] Grey ADNJ De Molecular Biology Intelligence Unit 9: The Mitochondrial Free Radical Theory of Aging. Austin, TX: R.G. Landes Company (1999).

[85] Weinstein RS Wan C Liu Q Wang Y Almeida M O'Brien CA. Endogenous glucocorticoids decrease skeletal angiogenesis, vascularity, hydration, and strength in aged mice. Aging Cell (2010) 9:147–61. 10.1111/j.1474-9726.2009.00545.x 20047574 PMC2858771

[86] Raff H Raff JL Duthie EH Wilson CR Sasse EA Rudman I. Elevated salivary cortisol in the evening in healthy elderly men and women: correlation with bone mineral density. J Gerontol Series A Biol Sci Med Sci. (1999) 54:M479–83. 10536652 10.1093/gerona/54.9.m479

[87] Reynolds RM Dennison EM Walker BR Syddall HE Wood PJ Andrew R. Cortisol secretion and rate of bone loss in a population-based cohort of elderly men and women. Calcif Tissue Int. (2005) 77:134–8. 10.1007/s00223-004-0270-2 16151676

[88] Dennison E Hindmarsh P Fall C Kellingray S Barker D Phillips D. Profiles of endogenous circulating cortisol and bone mineral density in healthy elderly men. J Clin Endocrinol Metab. (1999) 84:3058–63. 10.1210/jcem.84.9.5964 10487665

[89] Mcbride JR Fagerlund UHM Dye HM Bagshaw J Changes in structure of tissues and in plasma cortisol during the spawning migration of pink salmon, *Oncorhynchus gorbucha* (Walbaum). J Fish Biol. (1986) 29:153–66. 10.1111/j.1095-8649.1986.tb04934.x

[90] Zhang W Xu J Qiu J Xing C Li X Leng B. Novel and rapid osteoporosis model established in zebrafish using high iron stress. Biochem Biophys Res Comm. (2018) 496:654–60. 10.1016/j.bbrc.2017.12.172 29305866

[91] Carnovali M Luzi L Terruzzi I Banfi G Mariotti M. Metabolic and bone effects of high-fat diet in adult zebrafish. Endocrine (2018) 61:317–26. 10.1007/s12020-017-1494-z 29274064

[92] Carvalho FR Fernandes AR Cancela ML Gavaia PJ. Improved regeneration and de novo bone formation in a diabetic zebrafish model treated with paricalcitol and cinacalcet. Wound Repair Regener. (2017) 25:432–42. 10.1111/wrr.12536 28380670

[93] Fernández I Gavaia PJ Laizé V Cancela ML. Fish as a model to assess chemical toxicity in bone. Aquatic Toxicol. (2018) 194:208–26. 10.1016/j.aquatox.2017.11.015 29202272

